# Is the Influence of the Medical Profession on the Wane?

**Published:** 1890-01

**Authors:** 


### EDITORIAL DEPARTMENT.

F. E. DANIEL, M. D., EDITOR AND PUBLISHER.

THIS JOURNAL, by unanimous vote of the members, was made the Official Organ of the Austin District Medical Society.

COLLABORATORS:

*Dr. R. M. Swearingen, Austin.*
*Prof. B. E. Hadra, M. D., Galveston.*
*Prof. Geo. Cupples, M. D., San Antonio.*
*Dr. T. C. Osborn, Cleburne, Texas.*
*Dr E. J. Doering, Chicago.*
*Dr. E. J. Beall, Fort Worth.*
*Dr Odo Betz, Germany.*
*Dr. J. W. McLaughlin, Austin.*
*Dr. T. J. Tyner, Austin.*
*Prof. J. F. Y. Paine, M. D., Galveston.*
*Dr. R. H. L. Bibb, Mexico.*
*Dr. T. J. Bennett. Austin.*
*Dr. Bat Smith, Wharton.*
*Dr. E. Meierhof, New York.*
*Prof. W. B. Rogers, M. D., Memphis, Tenn.*

### IS THE INFLUENCE OF THE MEDICAL PROFESSION ON THE WANE?

A very few years ago one of the most prominent medical men of Texas,—a surgeon of note,—an ex-medical editor, said: "Texas medicine is an unknown quantity!— a terra incognito." At the risk of perpetrating what, in the vernacular, has become to be known as a "chestnut," we refer to the well-known slur of the British writer, "what good can come out of the Texas Nazareth?"

The N. Y. Medical Record, about three years ago, on reviewing a copy of the Transactions of the Texas State Medical Association, said, "Under the influence of spirited journalism affairs medical in Texas are assuming a more hopeful aspect; organization has been affected," etc. For several years the Transactions received most flattering notices from the medical press, and the profession of Texas was fairly installed in the foremost rank,—abreast of all sister States; the scientific ability of its members being conceded on all hands.

These recollections indicate the merging of the profession of medicine in Texas out of obscurity,—a rapid progress in professional favor, and even the attainment of eminence, in a short time; and the change has been ascribed to organization, under the stimulus of spirited journalism. When at the zenith of its fame,—the International Congress question came up. Those in charge failed to recognize the Texas profession, and would have utterly excluded it from representation in Congress, or participation in its organization. All will remember the indignant protest from Texas and the result; Texas medicine for once successfully asserted its rights, and they were respected.

In his Presidential address, Dr. F. W. Johns, in resigning the chair at the last meeting of the West Texas District Medical Association, said:

"But if I read correctly the signs of the times, the strength and patronage, the power and influence of the regular profession of medicine among the people is on the wane; and should we fail to stand shoulder to shoulder, county, district, State and nation, in the correction of the adverse conditions, the dissolution and downfall of your beloved organization is, at least, problematical."

Let us inquire into the truth of this bold assertion.

For years the Texas State Medical Association has petitioned the Texas Legislature, through committees of her ablest men, to enact a law to restrict the indiscriminate practice of medicine. In vain was it repeatedly pointed out that a large majority of the States of the Union had taken such steps; in vain were decisions of courts cited, declaring the constitutionality of such laws, and the competency of States to regulate the practice of medicine by statutory enactment. Every argument was exhausted in the endeavor to get even a modicum of justice in the way of such legislation.

Every petition failed; many of them were openly ridiculed.

The new capitol building contains many rooms for which no use can be found. Every department of the State government, every feature of social industry, of art and science,—law,—education,—geological,—historical,—statistics of insurance, agriculture, imigration, etc., is represented and furnished with elegant offices, furnished throughout, at public cost,—except the medical and sanitary science. There is absolutely no provision made for the preservation of even scraps of medical history, statistics of epidemics, or other diseases; no record of vital and mortuary statistics; no recognition whatever of the claims of the medical profession to representation as an integer of population, much less of government; and when the State Medical Association appointed a committee of its best men to petition the Legislature to allow them the use of one room for the preservation of its archives, and for a State Medical library, to be provided without cost to the State, and offered to furnish its own office, while all others are furnished at public cost; when this respectable committee waited on the Legislature in person, and urged their modest request, setting forth the importance of preserving medical history, and especially vital and mortuary statistics, stating that the society numbered five hundred members, embracing many educated men, and the best citizens, graduates of reputable colleges;—and that this membership represented a constituency of perhaps five thousand practitioners; what was the result? Not only was the request refused, but ridiculed in the most unblushing manner! A Senator, (God save the mark,) said, if the Doctors are to be provided with free quarters in the capitol, he would move for a similar provision for all other organizations; and cited the "Barbers" and "Boot-blacks."

Comment is unnecessary. The profession of medicine has never commanded the respect of the State; has never been recognized as an entity. How be it the scientific work of individual members as tabulated by the able and venerable Cupples, has received the plaudits of Europe's ablest surgeons, and the European medical press.

We come now to enquire why it is that the profession of medicine has failed to establish an influence with its own people; to enquire what are those "adverse conditions" which President Johns says should be corrected?

The people narrowly watch the meetings of the organized profession; most of its literature is read by many laymen. They realize that the standard of education, and of membership, is too low, and take note of the internal dissensions and unseemly disputes in the ranks. They have seen professional character assailed, and as clearly as any other have discerned through the transparent pretext, unworthy motives, perhaps malice, jealousy or revenge. No one more clearly than they recognize the presence of black sheep in the flock; men who could not secure entrance into their local or home organizations, have been admitted to the fold of the mother society. Papers by members have been published which have been openly ridiculed throughout the country. There are members who cannot spell the simplest words in the English language: of this we as an editor, have had abundant ocular proof. Those who have read "The Open door to Quackery" can readily understand that a diploma is no longer an evidence of professional competency, moral character or even the possession of a rudimentary English education; and yet, a diploma is practically the only requirement for membership.

The knowledge of these things on the part of the people, brings the profession into disrepute; the loaf is leavened by an element of unworthiness, and all suffer for the delinquencies of the few.

We may "stand shoulder to shoulder" as Dr. John says, but so long as little or no discretion is exercised in the selection of members, little or no cognizance taken of questionable morals or defective education; so long as ignorant men are put in position or assume a position, to display their ignorance, so long will the profession of medicine be held in disrepute.

The JOURNAL has striven for a higher education, a higher standard of professional character; for the elevation, purification and advancement of the brotherhood of medicine; and in organization, hoped to find the means to ends;—but,—alas, the fact stares us in the face, that though organized; though the ranks blaze with the glories of many brilliant escutcheons; though there are philosophers and scholars, physicians and surgeons of unquestioned ability and of unblemished life and character in the band, there is, as stated, an element of unworth which degrades the whole; asses' ears have been made painfully conspicuous, protruding through the lion-skin of office—in more instances then one,—aye, even from the presidential chair.

These are bitter words; but as the surgeon must cut away diseased tissue for the preservation of the body, so must the JOURNAL which aspires to elevate the profession of medicine and to "purify" its body, point out in plain terms those things which hinder and retard the work in hand. True it is, and "pity 'tis 'tis true," and it pains us to write it. But we should be recreants to our duty were we deterred by sentimentality, or fear of offending, from indicating needed reforms in the organization of the medical profession. We had hoped that when the Constitution and By-laws were in hand for revision, some change would be made whereby entrance into the organization would not be confined alone to the possession of a diploma.

